# Everybody's Page

**Published:** 1891-12-05

**Authors:** 


### Everybody's Page.

The word "demonstrative" was recently defined by a pupil teacher as "Showing in particular" like "a great beautiful blue golf suit"! Education is evidently making rapid strides. Another intelligent aspirant after knowledge asserted that, "At noon in December the sun disappears suddenly, but in June it can be seen to set below the horizon," an assertion which displays a remarkable amount of observation.

It is an incontrovertible fact, we believe, that many schools in the country under government inspection have neither cloak-rooms nor lavatories. Where the latter are wanting it is absolutely impossible to promote habits of cleanliness, and it is little wonder that there are so many insanitary dwellings if our children are brought up in the midst of surroundings that are a slur upon our vaunted civilisation. The atmosphere of these schools on wet days when the air is polluted by dirty caps and coats can better be imagined than described.

Small-pox is asserted to have been unknown in Mexico before the beginning of the sixteenth century, and it is said that a negro imported it in 1520. It soon spread over the country, claiming amongst its victims Cuitlahuatzin, Montezuma's brother, and the last but one of the emperors of the Aztecs. The disease in this part of the world appears to be most common in the spring and at the beginning of summer, more particularly in the temperate and cold zone than in the hot country. In the latter vaccination, which has only recently been obligatory in the federal district, and which is not generally practised, takes with more difficulty. Scarlet fever is very rare.

A writer in the *Therapeutic Gazette* protests against the practice of administering alcoholic stimulant of any kind to a patient suffering from hemorrhage, under the false idea of sustaining the vital power. The action of alcohol under such circumstances he protests against as injurious in every way. It excites the patient, relaxes the arteries, and favours the escape of blood through the divided structures, and above all other mischiefs increases the action of the heart, stimulating it to throw out more blood through the divided vessels. In every instance of syncope from loss of blood, so long as a patient could swallow, he would administer warm milk and water freely.

According to the *Pharmaceutical Journal of Australasia*, Baron Sir F. Von Mueller has introduced into Victoria the use of green Eucalyptus branches in sick rooms. He recommends the placing of them under bedsteads as applicable to all infectious and contagious diseases. Their use in this manner is alleged to have been successful with phthisical patients, both antiseptically and as a sedative, or even hypnotic in some cases. The experience of another physician, after a trial of twelve months, is stated to have been, that in scarlet fever the bedding is thus thoroughly disinfected, the volatile vapour penetrating the mattress as well as every other article, so that no other disinfection is required for the room, as the vapour destroys every germ. The remedy certainly has the great merit of simplicity.

The Municipal Commissioner of Baroda has published a pamphlet, in which he advocates the inoculation of the blood serum of the common weasel as a cure of snake-bite. This animal is, he contends, proof against the poison of snake-bites, from which it never suffers in the slightest degree, and attacks and kills any snake it comes across. Whether this is sufficient to constitute an effective antidote against a snake's venom has not yet, we believe, been proved by sufficient experience.

Schoolboys are often most humorous when least intending to be so. In a school under Government inspection the following sentence was given for correction:

"This England never did, nor never shall
Lie at the proud foot of a conqueror."

No doubt several boys rose to the occasion, but some did not, at least not in the way desired by the examiner. One candidate for scholastic fame stated that "foot should be feet, for a conqueror would have two feet." Another, "a foot cannot be proud, the word is misplaced." Yet another, "The sentence is wrong, for England *has* lain at the foot of a conqueror, viz., William the Conqueror." The inspector seemed to think these answers showed great want of intelligence, but we think the author of the last reply deserves a putty medal.

Since the introduction of the "safety" bicycle, cycling has undoubtedly increased in popularity. The result of this form of exercise on future generations has recently been foreshadowed in *Punch*. It is certainly an exception to see an erect rider with a good seat, the rule to see round shoulders and back bent nearly double. So much is this the case, that the question of the use of the machine is becoming an important hygienic problem. Some maintain that its use only develops muscles of the arms and legs, which any boy who indulges in ordinary athletic exercises develops, and, in addition, is the frequent cause of contracted chest and round shoulders. To a certain extent this cannot be denied, and it is important that all boys, when learning to ride should be taught how essential it is for their proper physical development that they should sit erect on their bicycle. With proper use, no harm should be done even to growing boys.

The sufferings of the poor Russians when migrating from one part of the Empire to another are asserted to be very terrible. They travel up the rivers in barges, which are much over-crowded. A writer in the *Northern Messenger* says: "I sometimes went in and cast a glance at these third class passengers, but hands, arms, legs, feet, heads, boots, bast shoes, sheepskins, sacks and bags with clothes, and dry bread, disclosed themselves, to my view, as one formless mass. I heard the helpless moans of sick children, one of whom was down with the small-pox; the atmosphere was fetid to an intolerable degree, it would positively knock down a person coming straight in from the fresh air. Coffins are left behind at every landing place containing the remains of peasants who have departed to the Elysian fields, and scores of children infected with small-pox, with diphtheria, with typhoid, are continually arriving at Tomsk, and augmenting the number of graves."

